# Patellofemoral arthroplasty for symptomatic nonunion after trochlear osteotomy for patellar instability: a case report

**DOI:** 10.1186/1757-1626-2-9086

**Published:** 2009-11-25

**Authors:** Hans-Peter W van Jonbergen, Kees van Egmond

**Affiliations:** 1 Department of Orthopaedic Surgery, Deventer Hospital, Nico Bolkesteinlaan 75, 7416 SE Deventer, The Netherlands; 2 Department of Orthopaedic Surgery, Isala Clinics, Groot Weezenland 20, 8011 JW Zwolle, The Netherlands

## Abstract

**Introduction:**

Patients with patellofemoral instability with trochlear dysplasia may be treated with trochlear osteotomy.

**Case presentation:**

We present a patient with patellofemoral instability treated with trochlear osteotomy. The procedure resulted in nonunion with painful bony impingement and isolated patellofemoral osteoarthritis. Patellofemoral arthroplasty was performed.

**Conclusion:**

Patellofemoral arthroplasty may be considered a salvage procedure for failed surgical treatment for trochlear dysplasia.

## Introduction

Patients with patellofemoral instability with trochlear dysplasia may be treated with trochlear osteotomy.

We report a case of a patient with symptomatic bony patellofemoral impingement after a failed trochlear osteotomy. She was subsequently treated with patellofemoral arthroplasty.

## Case presentation

**Figure 1:**
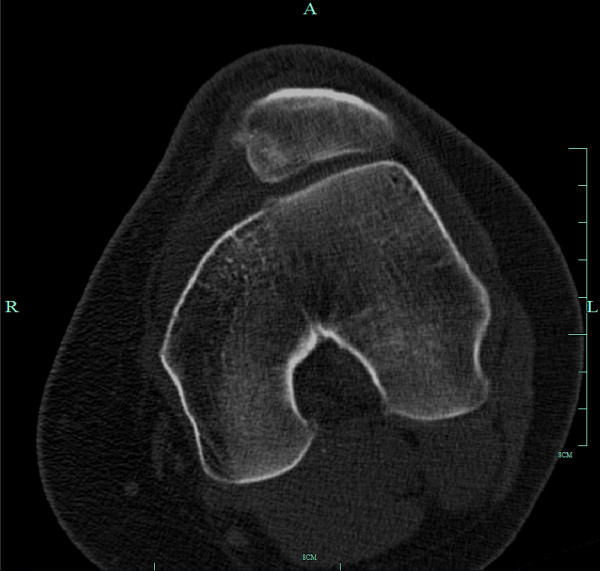
**Preoperative axial CT of the left knee showing trochlear dysplasia**.

A 33-year-old Dutch Caucasian woman was referred to our clinic with signs of painful bony impingement of the left patellofemoral joint. One year before presentation, she underwent a trochlear osteotomy of the left knee for recurrent patellofemoral instability with signs of trochlear dysplasia (Figure 1). There was no patella alta. Conservative treatment with physical therapy, activity modification, and taping had failed to relieve the patellofemoral instability. There was no history of any previous surgical procedures or trauma. In reviewing the operative records no complications during or directly after trochlear osteotomy were noted. Trochlear osteotomy was performed through a lateral parapatellar approach with the knee in full extension, allowing the patella to be retracted medially. After incomplete subchondral osteotomy of the anterior aspect of the lateral condyle with curved osteotomes, the articular cartilage of the anterior aspect of the lateral condyle was elevated in one piece to a height of 8 mm. Height was maintained by inserting a wedge shaped bone graft from the iliac crest and osteosynthesis with 2 screws. Patellofemoral stability was assessed through a full range of motion, and considered adequate. The synovial lining and lateral retinaculum were closed. No additional medial soft tissue surgery was performed. Direct postoperative partial weight bearing was advised with crutches, after 6 weeks full weight bearing was allowed.

**Figure 2:**
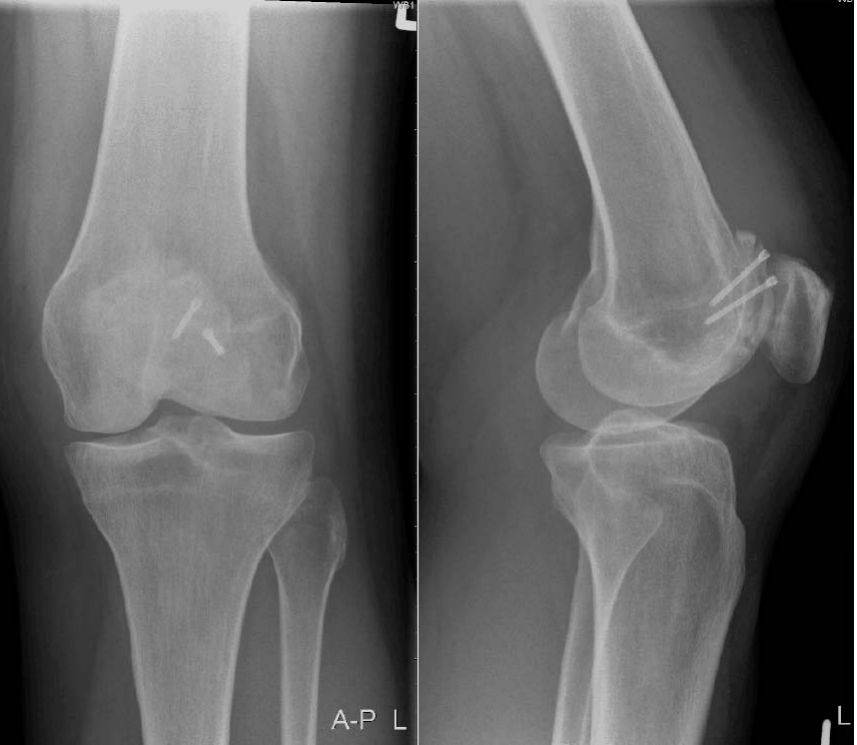
**Radiograph of the left knee 5 months after trochleaplasty**.

Five months later she still had persistent pain on the anterior aspect of the left knee without a history of trauma or patellar dislocation since the procedure. Radiographic examination demonstrated nonunion of the trochlear osteotomy with dislocation of subchondral bone resulting in an irregular patellofemoral joint (Figure 2). Arthroscopy was performed with removal of the screws. Since patellofemoral joint preserving surgery was considered inadequate, she was referred to our hospital for further treatment.

**Figure 3:**
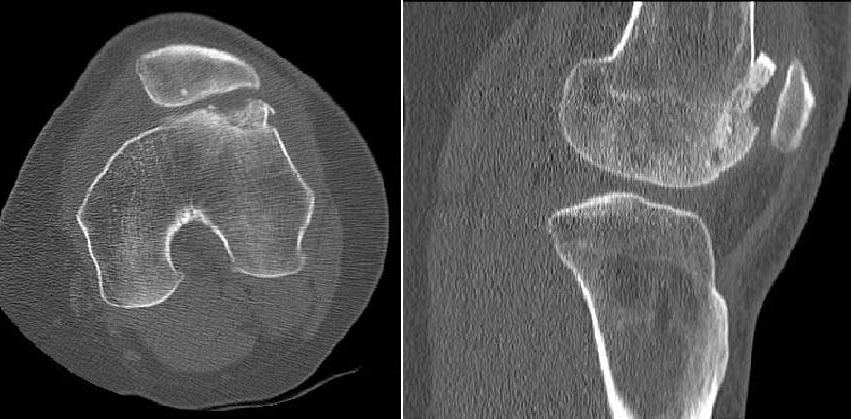
**Axial and lateral CT of left knee one year after trochleaplasty demonstrating irregular lateral condyle due to nonunion and collapse of subchondral bone**.

On physical examination there was painful limited range of motion with 90 degrees of flexion. No signs of infection were observed. There was obvious quadriceps muscle wasting. Palpation of lateral retinaculum and lateral condyle was painful. Moving from flexion to maximal extension, the patella was found to wobble at approximately 30 degrees of flexion. A Computed Tomography (CT) scan of the left knee demonstrated the irregular condyle (Figure 3).

**Figure 4:**
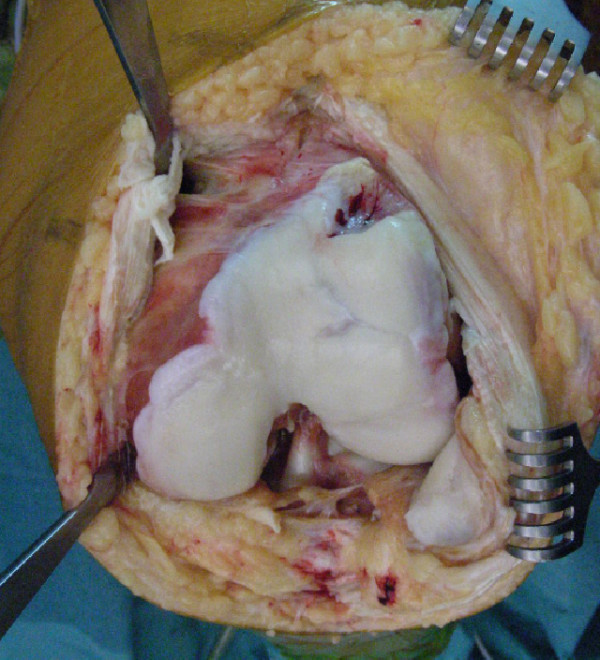
**Intra-operative photograph of the left knee**. Note the irregular lateral condyle.

**Figure 5:**
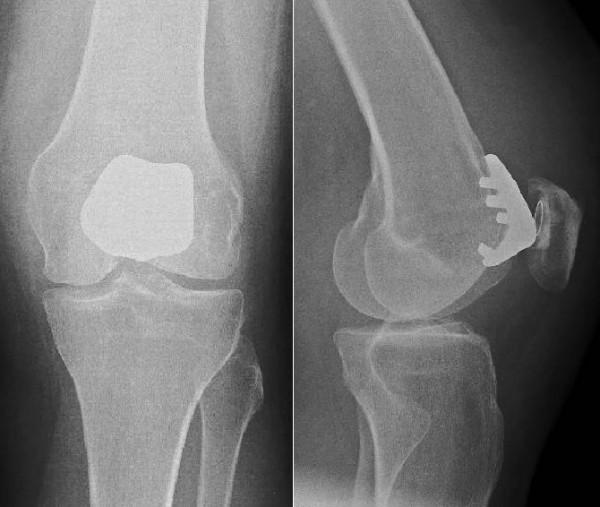
**Postoperative radiographs of the left knee with patellofemoral prosthesis in situ**.

Because of incapacitating pain, patellofemoral joint replacement was advised. Using a medial parapatellar incision, the knee joint was inspected. The anterior aspect of the lateral condyle was deformed with grade 4 cartilage degeneration. The remaining trochlea was dysplastic (Figure 4). Inspection of the patella showed no clear medial and lateral facets, with only the odd facet readily visible. On the lateral side a 1 × 1 cm grade 4 lesion was present. The femorotibial compartments showed no degenerative changes. Patellofemoral arthroplasty was performed using the Journey PFJ (Smith&Nephew, Memphis, Tennessee) (Figure 5). The irregular anterior femur was resected using the standard cutting blocks. Condylar support for the prosthesis was adequate. Postoperative rehabilitation was uneventful, and at last follow-up at 6 months the patient demonstrated good function of the left knee with 140 degrees of flexion. There were no signs of patellofemoral instability and she experienced only slight anterior knee pain.

## Conclusion

Patellofemoral instability may result from anatomical deficiencies in one of three anatomical structures that stabilise the patellofemoral joint: the trochlear groove geometry, the medial and lateral retinacula including the medial patellofemoral ligament (MPFL), and the alignment of the extensor apparatus including the quadriceps muscles, patellar tendon and tibial tuberosity. If nonoperative treatment fails, surgical treatment should aim to correct the identified anatomical deficiencies [1].

Trochleaplasty for recurrent patellar dislocation with associated trochlear dysplasia was first described in 1994 by Bereiter and Gautier [2]. The surgical procedure consists of chiselling off the medial and lateral condyles together with the trochlea as one piece in a proximal to distal fashion [3]. The osteochondral part remains fixed to the femur through a distally pedunculated flake. The trochlear groove is then deepened, and the osteochondral flap is seated and fixed in the newly created groove. Further soft tissue procedures aimed at enhancing patellofemoral stability may be required. Recently, good long-term results have been reported, with no patients experiencing recurrence of dislocation [3,4]. However, the outcome with respect to patellofemoral pain was less predictable. In the series described by Von Knoch et al. pre-operative pain was present in 78% of knees, and became worse in 33% of knees after trochleaplasty [3]. The reported complications include patella baja and a tendency for patellar subluxation.

Alternatively, with trochlear osteotomy only the lateral trochlea is incompletely osteotomized and levered to raise the lateral articular surface 6-8 mm [1]. The elevation of the lateral osteotomy is maintained by inserting a wedge shaped bone graft from the iliac crest with or without additional osteosynthesis. Koëter et al. reported satisfactory 4-year follow-up results in 17 knees [1]. Complications included subluxations in a patient with generalized hyperlaxity, and persistent pain in a patient who was subsequently treated with patellofemoral arthroplasty. Others noted a high complication rate with 4 of 5 patients significantly improved after 7-years follow-up [5].

Our patient had had a trochlear osteotomy with non-union and probably fracture of the osteochondral flake resulting in a painful patellofemoral joint with osteoarthritis. Because of the deformation of the lateral condyle, patellofemoral joint preserving surgery was considered inadequate. Total knee arthroplasty was considered too aggressive a treatment for what is, in effect, a disease confined to the anterior compartment. Patellofemoral arthroplasty is a successful treatment option for patients with isolated patellofemoral osteoarthritis, with long-term outcomes in young patients similar to those achieved in older patients [6].

We consider patellofemoral arthroplasty a salvage procedure for failed surgical treatment for trochlear dysplasia.

## List of abbreviations

CT: Computed Tomography; MPFL: Medial patellofemoral ligament; PFJ: patellofemoral joint.

## Competing interests

The authors declare that they have no competing interests.

## Authors' contributions

HPWvJ wrote the case report with input from KvE, KvE critically revised the manuscript. All authors have read and approved the final manuscript.

## Consent

Written informed consent was obtained from the patient for publication of this case report and accompanying images. The copy of the written consent is available for review by the Editor-in-Chief of this journal.
